# Prospective target assessment and multimodal prediction of survival for personalized and risk*-*adapted treatment strategies in multiple myeloma in the GMMG-MM5 multicenter trial

**DOI:** 10.1186/s13045-019-0750-5

**Published:** 2019-06-26

**Authors:** Dirk Hose, Susanne Beck, Hans Salwender, Martina Emde, Uta Bertsch, Christina Kunz, Christoph Scheid, Mathias Hänel, Katja Weisel, Thomas Hielscher, Marc S. Raab, Hartmut Goldschmidt, Anna Jauch, Jérôme Moreaux, Anja Seckinger

**Affiliations:** 1 Labor für Myelomforschung, Universitätsklinikum Heidelberg, Heidelberg, Germany; 2 Medizinische Klinik V, Universitätsklinikum Heidelberg, Heidelberg, Germany; 3 Department of Internal Medicine II, Asklepios Klinik Altona, Hamburg, Germany; 4 Deutsches Krebsforschungszentrum, Abteilung für Biostatistik, Heidelberg, Germany; 5 Department I of Internal Medicine, University of Cologne, Cologne, Germany; 6 Department of Internal Medicine III, Klinikum Chemnitz GmbH, Chemnitz, Germany; 7 Department of Hematology, Oncology and Immunology, University of Tübingen, Tübingen, Germany; 8 Nationales Centrum für Tumorerkrankungen, Heidelberg, Germany; 9 Universität Heidelberg, Institut für Humangenetik, Heidelberg, Germany; 10 IGH, CNRS, University of Montpellier, Montpellier, France; 11 Department of Biological Hematology, CHU Montpellier, Montpellier, France

**Keywords:** Multiple myeloma, Risk, Metascoring, Reporting, Survival

## Abstract

**Background:**

Personalized and risk-adapted treatment strategies in multiple myeloma prerequisite feasibility of prospective assessment, reporting of targets, and prediction of survival probability in clinical routine. Our aim was first to set up and prospectively test our experimental and analysis strategy to perform advanced molecular diagnostics, i.e., interphase fluorescence in-situ hybridization (iFISH) in ≥ 90% and gene expression profiling (GEP) in ≥ 80% of patients within the first cycle of induction chemotherapy in a phase III trial, seen as prerequisite for target expression-based personalized treatment strategies. Secondly, whether the assessment of risk based on the integration of clinical, cytogenetic, and expression-based parameters (“metascoring”) is possible in this setting and superior to the use of single prognostic factors.

**Methods:**

We prospectively performed plasma cell purification, GEP using DNA-microarrays, and iFISH within our randomized multicenter GMMG-MM5-trial recruiting 604 patients between July 2010 and November 2013. Patient data were analyzed using our published gene expression report (GEP-R): after quality and identity control, integrated risk assessment (HM metascore) and targets were reported in clinical routine as pdf-document.

**Results:**

Bone marrow aspirates were obtained from 573/604 patients (95%) and could be CD138-purified in 559/573 (97.6%). Of these, iFISH-analysis was possible in 556 (99.5%), GEP in 458 (82%). Identity control using predictors for sex, light and heavy chain type allowed the exclusion of potential sample interchanges (none occurred). All samples passed quality control. As exemplary targets, IGF1R-expression was reported expressed in 33.1%, AURKA in 43.2% of patients. Risk stratification using an integrated approach, i.e., HM metascore, delineated 10/77/13% of patients as high/medium/low risk, transmitting into significantly different median progression-free survival (PFS) of 15 vs. 39 months vs. not reached (NR; *P* < 0.001) and median overall survival (OS) of 41 months vs. NR vs. NR (*P* < 0.001). Five-year PFS and OS-rates were 5/31/54% and 25/68/98%, respectively. Survival prediction by HM metascore (Brier score 0.132, *P* < 0.001) is superior compared with the current gold standard, i.e., revised ISS score (0.137, *P* = 0.005).

**Conclusions:**

Prospective assessment and reporting of targets and risk by GEP-R in clinical routine are feasible in ≥ 80% of patients within the first cycle of induction chemotherapy, simultaneously allowing superior survival prediction.

**Electronic supplementary material:**

The online version of this article (10.1186/s13045-019-0750-5) contains supplementary material, which is available to authorized users.

## Background

Multiple myeloma is a malignant hematological disease characterized by accumulation of clonal plasma cells in the bone marrow and associated clinical signs and symptoms, especially those related to the displacement of normal hematopoiesis and generation of osteolytic bone disease [1].

Introduction of high-dose melphalan followed by autologous stem cell transplantation, proteasome inhibitors, immunomodulatory drugs, and monoclonal antibodies improved survival [2–5] without curing a significant fraction of patients. Prognosis of individual patients is highly heterogeneous: current treatment algorithms do well for some patient groups with a median survival of more than 10 years, while a significant proportion of patients shows median survival of 2 years or even below [6, 7]. This frequently prompts the suggestion to treat the latter patients differently, most often more aggressively, in other terms to perform risk-adapted treatment, e.g., UAMS total therapy [8], mSMART by the Mayo Clinic [9], or the GMMG-CONCEPT trial (NCT03104842).

Risk stratifications in clinical routine are usually performed by combination of presence of high-risk chromosomal aberrations as detected by interphase fluorescence in-situ hybridization (iFISH) and the International Staging System (ISS) as exemplified by the revised ISS score (rISS) and others [10–12]. At the same time, prognostic power can be increased by assessing gene expression, i.e., proliferation [13] and high risk scores [14–20], e.g., by DNA-microarrays (gene expression profiling, GEP). This is, however, rarely used prospectively in clinical routine or in a clinical trial setting. In turn, ending up with a variety of clinical and molecular prognostic factors, it is necessary to integrate different factors into a single prognostic information (metascoring).

Besides risk stratification, assessment of gene expression allows investigation of aberrantly or overexpressed targets exemplified by Aurora-kinase A (AURKA) [21] or insulin-like growth factor 1 receptor (IGF1R) [22]. Expression of these targets, in turn, is associated with adverse survival [21, 22]. For potential personalized treatment, the addition of clinical grade inhibitors to a backbone treatment only in those patients whose myeloma cells actually express the respective target could be envisioned. Whereas the number of actionable targets in multiple myeloma is currently largely limited, it is very likely that the advent of immunotherapy will change this.

Both personalized and risk-adapted treatment strategies prerequisite the feasibility of prospective assessment and reporting of targets and prediction of survival probability in clinical routine in a high enough percentage of patients. For phase III trial strategies, e.g., selecting an add-on treatment, the necessary threshold based on power calculations and clinical feasibility could be estimated as 80% of the actual population of patients included in the trial. It is interesting to denote that, despite of course iFISH and GEP have been used in an academic setting or using commercial providers [8, 17, 23–28], the question has yet not been answered if this is possible in terms of a *prospective molecular analysis and reporting*, as opposed to being conducted in a prospective clinical trial. Our study group sees such a proof as prerequisite for indeed planning a clinical trial based on molecular diagnostics such as GEP or RNA-sequencing.

Aims of this study were thus: (i) set up a sampling, experimental and analysis strategy to perform iFISH in ≥ 90% and GEP in ≥ 80% of patients within the first cycle of induction chemotherapy. (ii) Report to patients and physicians within this time to be able to draw a clinical consequence. (iii) Prospectively validate this strategy in the randomized phase III multicenter GMMG-MM5-trial including assessment of potential targets (based on GEP) and multimodal assessment of risk using clinical, cytogenetic, and gene expression-based prognostic factors and their integration into a metascore in clinical routine.

## Methods

### Patients

Six hundred and four patients were included in the prospective, open-label, randomized multicenter phase III clinical trial (EudraCT no. 2010-019173-16) between July 2010 and November 2013. A total of 31 transplant centers and 75 associated sites throughout Germany are participating in this trial initiated by the GMMG and approved by ethics committees of the University of Heidelberg and all participating sites. The MM5 trial was conducted according to the European Clinical Trial Directive (2005) and the Declaration of Helsinki. Patients were equally randomized to each of the four treatment arms (A1, A2, B1, and B2) using block randomization, stratified by ISS stage. Treatment consisted of either three 4-week cycles of PAd (A1+B1) or three 3-week cycles of VCD (A2+B2). Thereafter, standard intensification according to local protocols (GMMG standard) was performed, including stem cell mobilization and leukapheresis followed by single high-dose therapy or, for patients not achieving near complete response (nCR) or better, tandem high-dose therapy. Subsequently, consolidation therapy consisting of two cycles of lenalidomide (25 mg, days 1–21) followed by lenalidomide maintenance (for the first 3 months, 10 mg/day continuously and thereafter 15 mg/day continuously) for either 2 years (A1+A2) or until CR (B1+B2) was applied. Patients were followed until April 2017. Trial results regarding the primary endpoint, i.e., non-inferiority of VCD vs. PAd induction treatment have already been published [29], or are currently under review. With written informed consent as depicted above, patients were simultaneously included in the pre-planned prospective conducting of advanced molecular diagnostics, i.e., iFISH and GEP.

### Sampling strategy

Sixty to 80 ml of heparinized bone marrow (i.e., 3–4 × 18 ml of bone marrow plus 2 ml of heparin per syringe, aspirated with three bone marrow punctures within one anesthetized region) were drawn before the start of chemotherapy and sent to the Multiple Myeloma Research Laboratory Heidelberg by overnight express. For in-house samples, technicians of the Multiple Myeloma Research Laboratory attended the bone marrow aspiration providing assistance to the physician and allowing the fastest possible processing of the samples. Plasma cell purification including quality control and preparation of samples for iFISH (i.e., cytospins) and GEP (see below for details) were performed centrally according to our laboratory SOP immediately after the arrival of the sample. Sample submitting centers were informed electronically about the quality of the aspirate and the results of the plasma cell purification.

### Purification of CD138^+^ plasma cells

Density gradient centrifugation of bone marrow aspirates over Ficoll Hypaque (Biochrom, Berlin, Germany) was performed to separate mononuclear cells by standard protocol. CD138^+^ plasma cells were isolated using anti-CD138 immunobeads and an autoMACS Pro Separator (Miltenyi Biotec, Bergisch Gladbach, Germany) as published [13, 21, 30–35]. Purity was assessed by flow cytometry (Becton Dickinson, Heidelberg, Germany) using antibodies against CD38 (clone HB-7, FITC-labeled; Becton Dickinson) and CD138 (clone B-B4, PE-labeled; Miltenyi Biotec). Aliquots of CD138^+^ malignant plasma cells were subjected to cytospin preparation with 5000 cells per dot for iFISH analysis (*n* = 556 patients) and RNA/DNA extraction for gene expression profiling (*n* = 458).

### Interphase fluorescence in situ hybridization

iFISH analysis was conducted on CD138-purified plasma cells using probes for numerical changes of the chromosome regions 1q21, 5p15, 5q31 or 5q35, 8p21, 9q34, 11q22.3 or 11q23, 13q14.3, 15q22, 17p13, and 19q13, as well as translocations t(4;14)(p16.3;q32.3), t(11;14)(q13;q32.3), and t(14;16)(q32.3;q23) or any other IgH rearrangement with unknown translocation partner, according to the manufacturer’s instructions (Kreatech, Amsterdam, The Netherlands and MetaSystems, Altlussheim, Germany) and data were analyzed as published [36].

### Analysis of gene expression

RNA was extracted using the Qiagen AllPrep DNA/RNA kit (Qiagen, Hilden, Germany) according to the manufacturer’s instructions. Quality control and quantification of total RNA was performed using an Agilent 2100 bioanalyzer (Agilent, Frankfurt, Germany).

Gene expression profiling using U133 2.0 plus arrays (Affymetrix, Santa Clara, CA, USA) was performed as published [13, 32, 33]. Expression data are deposited in ArrayExpress under accession number E-MTAB-2299.

### Reporting of GEP-R

Our gene expression report (GEP-R) [31] is a non-commercial software framework developed within the open source software environments R [37] and Bioconductor [38] that can be adapted to other parameters or disease entities. It includes classifications of myeloma, i.e., TC [39]-, EC [40]-, and molecular classification [41], risk stratification, i.e., UAMS GEP70 [14]- and IFM 15-gene score [15], and our gene expression-based proliferation index (GPI) [13], and assessment of target gene expression, e.g., for immunotherapeutic or individualized treatment approaches, into one report. The GEP-R runs a quality and identity control; the latter is based on prediction analysis for microarrays (PAM) [42] predictors for sex, IgL (lambda, kappa), and IgH type (IgA, IgG, IgD). Results are reported as pdf document consisting of a two pages report given to the treating physician and an appendix containing details regarding the quality and identity control, as well as the assessment of gene expression. Within the GEP-R, the implemented HM metascore integrates gene expression-based and conventional prognostic factors into one prognostic classification [31]. The HM metascore has already been validated by cross-validation and an external validation cohort [31].

### Statistical analysis

Effects were considered statistically significant if the P-value of corresponding statistical tests was below 5%. Overall (OS) and progression-free survival (PFS) were investigated using Cox’s proportional hazard model as published [43, 44]. Survival curves were computed with nonparametric survival estimates for censored data using the Kaplan-Meier method [45, 46]. Difference between the curves was tested using the G-rho Log-rank test [47]. A subset of 451 patients with complete prediction information was used within a 73 months period for evaluating the performance of the risk prediction models in OS analysis. Cox’s proportional hazard models were used as input; for cross-validation, subsampling parameter of 301 and bootstrapping parameter of 150 were chosen. The integrated Brier score was used to assess prediction accuracy [48–50]. For statistical testing, the van de Wiel test was used [50].

## Results

### Feasibility of sampling and plasma cell purification

**Fig. 1:**
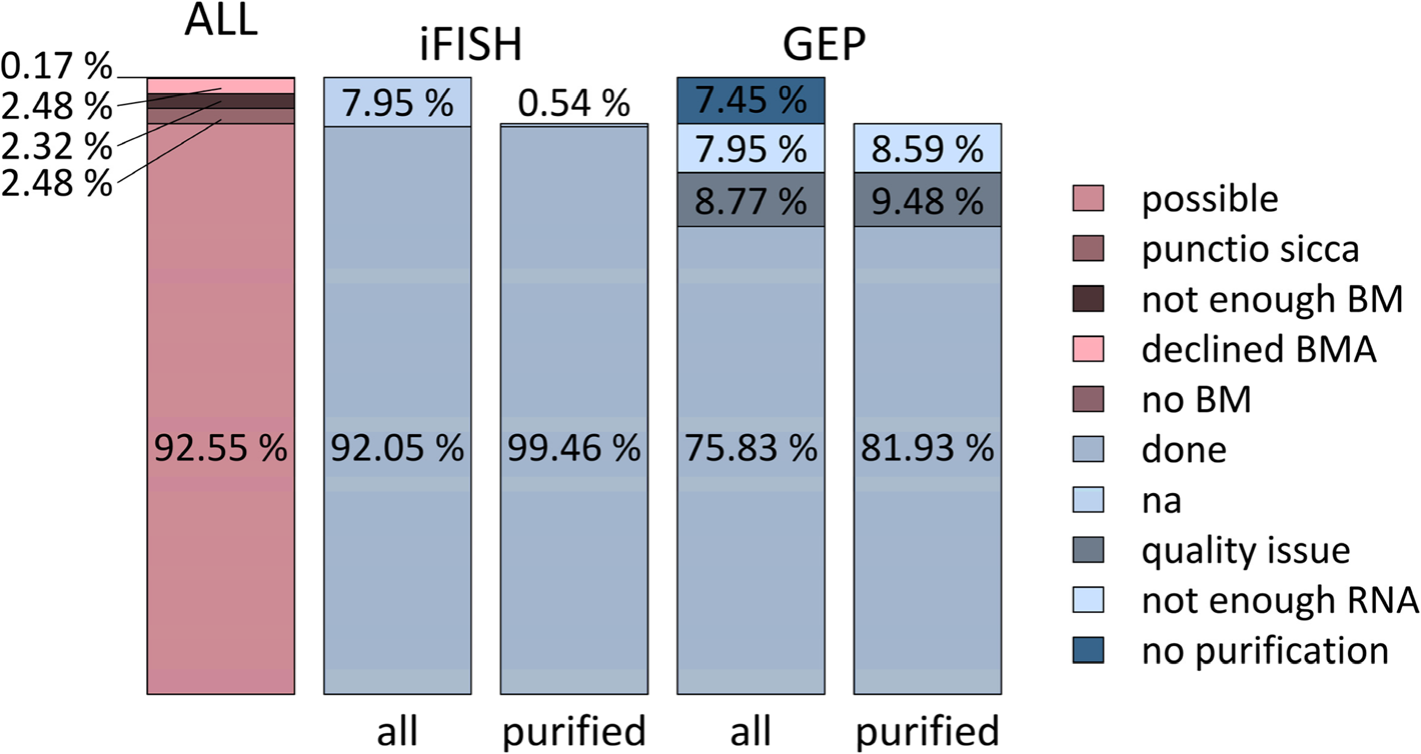
Feasibility of plasma cell purification, iFISH, and gene expression profiling within the GMMG-MM5 trial. Percentages are given for feasibility of plasma cell purification (ALL) as well as performing of interphase fluorescence in situ hybridization (iFISH) and gene expression profiling (GEP) using DNA-microarrays. For the latter two, columns refer to both all patients and those for which purified plasma cells were available. BM(A), bone marrow (aspiration); na, not available

Six hundred and four patients were included in the GMMG multicenter MM5-trial between July 2010 and November 2013, with a total of 31 participating transplant centers and 75 associated sites throughout Germany. In accordance with the pre-planned prospective protocol, bone marrow aspirates at the time of inclusion in the study, i.e., before start of treatment, were available for *n* = 573 patients (94.9%) with a median volume of 75 ml (standard deviation (SD): 23.4), of whom we were able to successfully perform plasma cell purification followed by quality control using flow cytometry for *n* = 559 patients (97.6%) according to our laboratory SOP. The 31 lacking samples (5.1%) were due to patients declining the bone marrow aspiration (2.5%) or *punctio sicca* (2.5%) (Fig. 1). Median purity according to CD38/CD138 double staining was 87.9% (SD 16.5%) with a median cell number of 1.2 × 10^6^ cells (SD 8.5 × 10^6^).

### Feasibility of iFISH and GEP

iFISH using cytospins from CD138-purified plasma cells was performed centrally for the trial (Multiple Myeloma Research Laboratory and Department of Human Genetics). Cytospins were prepared with 5000 cells per dot on the day of purification and hybridized immediately thereafter. Data could be obtained for 556/573 patients with available bone marrow aspirates (97%) and 556/559 patients with available CD138-purified plasma cells, respectively (99.5%; Fig. 1). The median proportion of malignant plasma cells as per iFISH, i.e., the highest percentage of a chromosomal aberration, was 95% (SD 20%).

Samples for RNA extraction followed by quality control were collected over 2 weeks each and then subjected to gene expression profiling by DNA-microarrays. In total, *n* = 458 transcriptome datasets are available; i.e., 81.9% of patients with available CD138-purified plasma cells. Of these, two patients were excluded from further analysis as they did not fulfill the inclusion criteria of the trial. Gene expression profiling could not be performed in 53 cases due to low RNA quality (9.5%) and further 48 cases (8.6%) in which not enough RNA was available (Fig. 1). Gene expression data were then analyzed and reported using our previously published GEP-R [31].

### Identity and quality control

In the 456 GEP-R from the intention-to-treat population, we exclude an interchange of samples using the implemented identity control. Predictors for light and heavy chain type (overall error rate 2.2% and 1.6%, respectively) as well as the sex of the patient (5.5% overall error rate, in agreement with frequent loss of the Y-chromosome [51]; being “female” can be predicted without error) showed comparable results in this prospectively analyzed cohort of patients if compared to the original publication (retrospectively on the validation cohort: 6%, 1%, and 4%, respectively [31]). False predictions were found in 3/93 patients with IgA myeloma (3.2%) and 3/269 patients with IgG (1%) as well as 4/308 patients with kappa light chains (1.3%) and 6/148 patients with light chains type lambda (4%; Additional file 1: Table S1). No sample failed in all three criteria.

As per the implemented quality control with a total of 7 quality parameters, the majority of patients showed no abnormality (339, 74.4%), 110 had a warning in one minor criterion (24.1%), 6 patients in 2 (1.3%), and 1 patient in 3 (0.2%). No patient fulfilled one of the major exclusion criteria based on previously performed QC analysis.

### Risk assessment and classifications of myeloma

Fifty-three patients were predicted using the GEP-R to have a t(4;14), corresponding to 11.6% of the total cohort with available GEP data. In five patients, iFISH and GEP showed discrepant results with the alteration being not found in iFISH. In conservative estimation, we considered the PAM-based predictor to have an overall error rate of 1.1%. Three of these patients had a clonal IgH-translocation with an unknown translocation partner, one patient had a clonal t(11;14), and one patient did not have a detectable translocation involving the IgH-locus. Predicted t(4;14) status delineated significantly different median PFS of 40 vs. 26 months (*P* = 0.008) and median OS of not reached (NR) vs. 56 months (*P* = 0.003; Additional file 2: Figure S1).

Regarding the GEP70 score, 113 patients were attributed as being high risk (24.8%) and 343 (75.2%) as low risk which transmitted into significantly different median PFS of 23 vs. 43 months (*P* < 0.001) and median OS of 41 months vs. NR (*P* < 0.001; Additional file 2: Figure S1). The same holds true for the IFM15 score with 106 patients being high risk (23.2%) and 350 patients (76.8%) being low risk transmitting into median PFS of 25 vs. 44 months (*P* < 0.001) and OS of 57 months vs. NR (*P* < 0.001; Additional file 2: Figure S1). Regarding myeloma cell proliferation, 36 (7.9%), 191 (41.9%), and 229 (50.2%) patients were attributed as being GPI^high^, GPI^medium^, and GPI^low^, respectively with significantly different median PFS of 18 vs. 33 vs. 49 months (*P* < 0.001) and median OS of 33 vs. 76 months vs. NR (*P* < 0.001; Additional file 2: Figure S1).

**Fig. 2:**
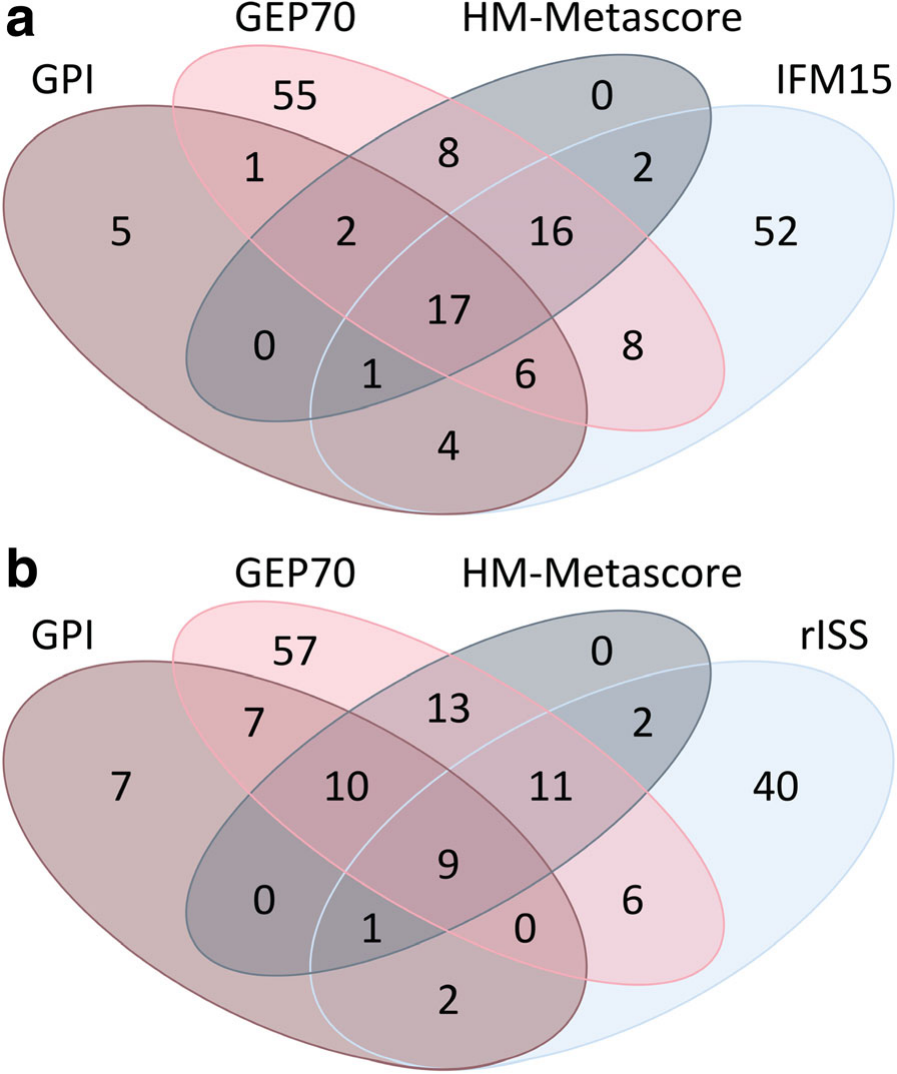
Overlap of patients identified as being of high risk by conventional prognostic factors, gene expression-based risk scores or proliferation, and metascores. Venn diagrams showing overlap of patients identified as being of high risk by **a** gene expression-based risk scores or proliferation, i.e., GEP70, IFM15 scores, and GPI, as well as HM metascore and **b** GEP70, IFM15 scores, GPI, and rISS. See Table 1 for details and further prognostic factors. rISS, International Staging System; GPI, gene expression-based proliferation index

The percentages of patients identified as being of high risk by conventional prognostic factors, gene expression-based risk scores or proliferation (GPI), as well as metascores and the overlap of the respective groups of patients are shown in Table 1 and Fig. 2.

**Table 1:**
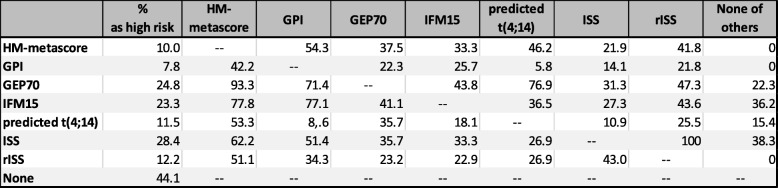
Delineation of “high-risk” patients by the respective variables and scores

The percentages of patients identified as being of high risk (first column) and the overlap of the respective groups of patients are shown

*GPI*, gene expression-based proliferation index; *(r)ISS*, (revised) International Staging System

For the results of grouping myeloma into different subentities, see Additional file 2: Figure S2.

### Target assessment

Expression height and presence/absence of expression were assessed for (i) examples of potential “target genes.” As the aim of our manuscript was prospective testing of the GEP-R, these examples include AURKA, FGFR3, and IGF1R, for which at that time potential clinical grade inhibitors were foreseen, (ii) potential targets for immunotherapy, i.e., cancer testis antigens like CTAG1, MAGE1, and HM1.24, and (iii) genes frequently aberrantly or differentially expressed in myeloma. In our cohort of 456 patients from the intention-to-treat population for which a GEP-R is available, 197 were found to express AURKA (43.2%), 151 IGF1R (33.1%), and 50 FGFR3 (11%), respectively. In the same way, candidates for personalized treatment approaches, given the availability of respective inhibitors, could be addressed.

## Metascoring

**Fig. 3:**
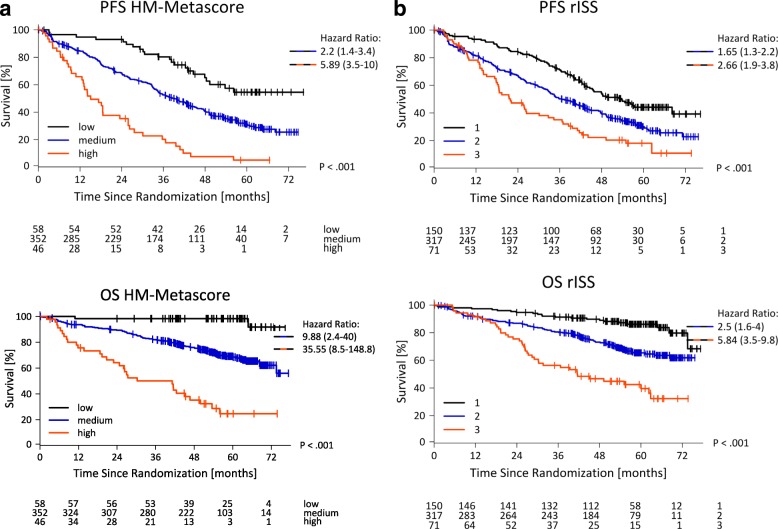
Prognostic impact of HM metascore and rISS. Shown are progression-free (PFS) and overall survival (OS) for **a** our HM metacore as well as **b** the revised International Staging System (rISS)

The HM metascore had already been validated on an external cohort as part of its initial set up [31]. In prospective testing, 58 myeloma patients were classified as being low risk (12.7%), 352 as medium risk (77.2%), and 46 were attributed as being high risk (10.1%). This transmitted into significant different median PFS of NR vs. 39 vs. 15 months (*P* < 0.001) and OS of NR vs. NR vs. 41 months (*P* < 0.001). At 5 years, survival rate was 98% vs. 68% vs. 25% and 57 (98.2%) vs. 253 (71.8%) vs. 15 (32.6%) patients were still alive (Fig. 3).

**Fig. 4:**
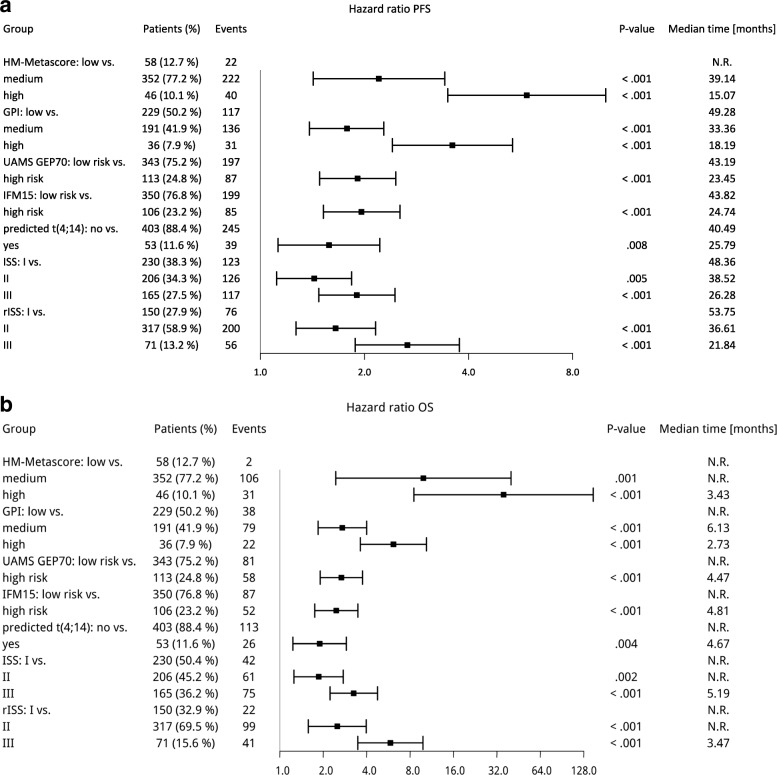
Forest plots. Forest plots summarizing the prognostic impact of individual factors and metascores (i.e., revised International Staging System (rISS) and HM metascore) in terms of **a** progression-free and **b** overall survival. GPI, gene expression-based proliferation index. The hazard ratios are shown on a log scale

Applying the rISS as the current gold standard, 150 patients were classified as being rISS I (27.8%), 317 as rISS II (58.9%), and 71 were attributed as rISS III (13.2%), respectively, transmitting into significant different median PFS of 54 vs. 37 vs. 22 months (*P* < 0.001) and median OS of NR vs. NR vs. 41 months (*P*<0.001). Five-year PFS and OS-rates for rISS I/II/III were 44/30/18% and 86/65/40%, respectively (Fig. 3). Forest plots summarizing the prognostic impact of individual factors and metascores (i.e., rISS and HM metascore) can be found in Fig. 4.

**Fig. 5:**
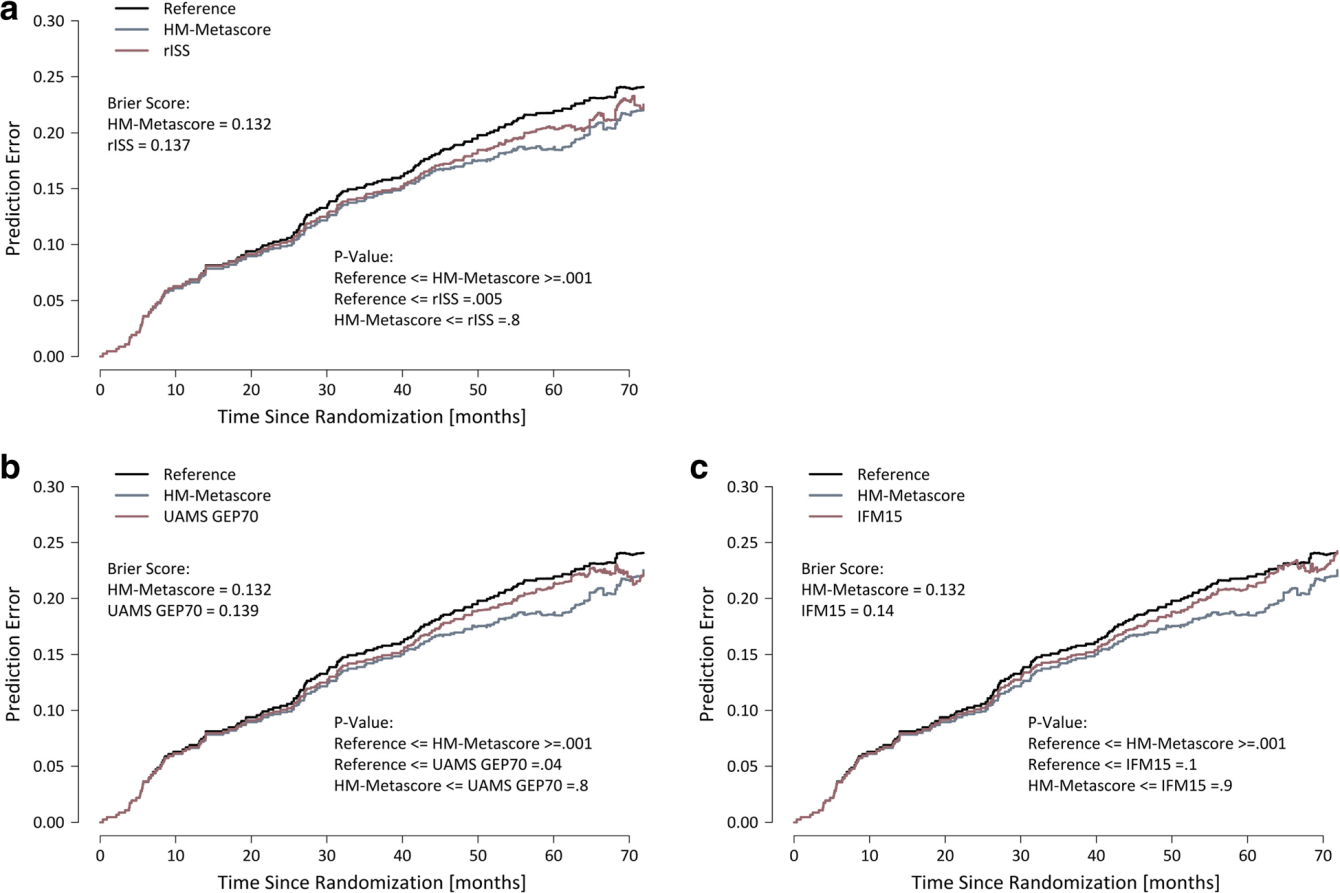
Brier score. Integrated Brier score assessing accuracy of prediction for HM metascore vs. **a** revised International Staging System (rISS), **b** GEP70 score, and **c** IFM15 score compared to the reference. Brier scores (prediction error) as well as *P* values for the different comparisons are given (bottom right, respectively) for overall survival are given

An integrated Brier score was calculated to assess prediction accuracy. The overall predictive value for OS from 0 to 73 months is best for the HM metascore (0.132) compared to rISS (0.137) or other GEP-based scores, i.e., the GEP70- (0.139), IFM15 models (0.14), or Reference (0.148), respectively (Fig. 5). Differences between the HM metascore and the other models were, however, not statistically significant. Likewise, differences between the “conventional” ISS and rISS were not significantly different.

## Discussion

Personalized and risk-adapted treatment strategies in multiple myeloma prerequisite the feasibility of prospective assessment, reporting of targets and prediction of survival probability in clinical routine—in a “high enough” percentage of patients. Besides, they also necessitate the availability of treatment options that can indeed be applied in a personalized manner (see below). Based on statistical power calculations for subsequent trials, we defined the thresholds as 90% for iFISH and 80% for GEP-based assessment.

### Can you do it in clinical routine?

Despite iFISH, GEP and RNA-sequencing have been used in academic setting or using commercial providers, to the best of our knowledge, no data is available regarding a *prospective molecular analysis and reporting* in clinical routine in a way that this could be used for personalized and risk-adapted treatment strategies, e.g., during the first cycle of induction chemotherapy. This is a different setting from being able to run advanced molecular diagnostics in a prospective trial in a subpopulation of patients which has frequently been shown by others and us [8, 17, 23–28].

In our GMMG-MM5 trial, consenting to bone marrow aspiration and molecular analyses was not a prerequisite to participate in the trial, in contrast, e.g., to the total therapy 4 and 5 trial with mandatory GEP data [8, 23], as it did not imply any up-front clinical consequences. Nonetheless, 95% of patients agreed to bone marrow aspiration at inclusion in the trial. This rate was driven by a high motivation of participating centers and physicians to explain patients planned analyses and the usefulness of translational research in multiple myeloma, and a concomitant intrinsic willingness of patients to participate. At the same time, we motivated participating centers by direct feedback of sample quality and result of purification, as well as iFISH and GEP (see below). The set-up of our sampling and analysis strategy allowed performing iFISH in 97% and GEP in 80% of patients with available bone marrow samples. This compares favorably to iFISH results reported by the EMN02/HOVON95-trial (74.1%) [52], IFM2009-trial (73.6%) [53], DSMM XI-trial (73.7%) [54], SWOG S0777-trial (60.2%) [55], or a pooled analysis of three PETHEMA/GEM clinical trials, i.e., GEM2000, GEM2005MENOS65, and GEM2010MAS65 (60.8%) [56], which were conducted during a comparable time frame. We have therefore for the first time validated prospectively in a randomized phase III multicenter trial the possibility to perform not only cytogenetic (including rISS) but also gene expression-based risk stratification and reporting in > 80% of patients during the first cycle of induction chemotherapy as—potentially—molecular risk-adapted, personalized treatment strategy.

### Why would you want to do it?

Besides risk stratification which can be done by both iFISH and GEP [20], the specific benefit of gene expression profiling, either by DNA-microarrays as in this trial or by RNA-sequencing, lies in the additional ability to identify target gene expression. In our analysis, this was intended for immunological targets and those for which small molecules or antibodies existed, e.g., Aurora-kinase A (VX-680 [21]), IGF1-receptor (e.g., AVE1642 [57]), or FGF3R (e.g., CHIR-258 [58]). AURKA was selected at this point in time when the GEP-R was developed as we had previously shown it to be expressed in approx. 30% of previously untreated myeloma patients and is associated with adverse survival [21]. IGF1R-inhibition was selected due to its importance as myeloma growth factor and impact on patient survival [22, 59]. As our aim was the prospective testing of our approach, we retained both factors also in the HM metascore, even given that neither will in 2019 be used as a clinical target. Here, it was our intention to give the proof-of-principle for prospective advanced molecular diagnostics of targets and reporting in clinical routine; in this way, the GEP-R is depicted and should be interpreted. Novel targets for which clinical grade inhibitors become available or immunological targets can be added to the assessment due to the adaptable surface of our reporting tool (GEP-R) [31]. Without a doubt, actual implementation necessitates standardization and either commercial or academic development of an actual molecular diagnostics test. Although this is beyond the scope of our manuscript, we show here that such a strategy is in principle feasible.

Alongside the principle possibility of running advanced molecular profiling and depiction of potential targets for individualized treatment, assessment of risk was the third main objective of our study. Here, many prognostic factors have been described with the ongoing discussion of which to include [19, 20]. This leaves the treating physician with a plethora of information that is difficult to consolidate intending counseling patients and drawing a clinical conclusion. Metascoring appears as an appropriate strategy [18, 31, 60, 61] to overcome this, and we show that this is likewise possible in a randomized clinical trial setting. Regarding the molecular techniques used in the metascore, i.e., iFISH and GEP, we choose to include both due to in part non-overlapping prognostic information, e.g., it is not possible to predict del17p13 at a high-enough accuracy by GEP [62].

The actual (good) prediction result of our metascore per se is thereby not the main focus of our analysis. Nonetheless, even with this “2010 choice” regarding risk factors and target genes, metascoring including GEP-based risk assessment is superior in numbers to rISS, although not statistically so. The same however holds true for a comparison of rISS to ISS even on our comparably large cohort of patients.

### Can we do better?

For a molecular diagnostics-based trial, bone marrow assessment, submission of bone marrow of sufficient quality and quantity are mandatory prerequisites. Based on our optimized protocol, data in terms of purity and cell number could be further improved within the prospective multicenter GMMG-HD6 trial (NCT02495922) recruiting 564 patients between June 2015 and September 2017 to a median purity of 94.5% (SD 13.2%) and 1.4 × 10^6^ (SD 34.8 × 10^6^) purified plasma cells. Future directions comprise the optimization of patient recruitment and sampling strategy in terms of mandatory bone marrow aspiration as inclusion criterion as defined above and replacement of GEP by RNA-sequencing, currently tested in our BMBF-funded CLIOMMICS-project. RNA-sequencing has several advantages over GEP using microarrays [63, 64]: (i) it provides quantification of levels of transcripts without significant saturation effects, (ii) it does not prerequisite a priori definition of sequences to be analyzed (as are, e.g., Affymetrix “probesets”) and thus allows detection of mutated transcripts, e.g., targetable BRAF-mutations. Likewise, transcripts, for which initially incorrect sequences were assumed, and thus corresponding probesets do not interrogate the transcript of interest, can be analyzed. (iii) RNA-sequencing enables the analysis of splice variants, as well as (iv) the investigation of other RNA types, for example, miRNAs [65], and (v) it can routinely be performed from as low input as 10 pg of total RNA compared to about 100 ng for microarrays and a double amplification protocol [63]. The latter is especially important, as the amount of RNA did not permit an analysis by GEP in approx. ten percent of patients in the GMMG-MM5 trial. Despite these advantages, there are also several caveats and challenges including data storage and handling due to the large size of RNA-sequencing raw datasets, time-consuming bioinformatics analysis, and less standardization [64, 66, 67].

## Conclusion

In conclusion, using an elaborated sampling, experimental and analysis strategy as reported here, we show for the first time that it is possible to prospectively perform and report molecular analyses in a randomized phase III multicenter trial in over 90% (iFISH) and 80% (GEP) of patients, respectively, within the first cycle of induction chemotherapy. Therefore, we validate that a trial strategy using either of the methods is possible, including reporting of potentially actionable targets. Risk assessment using our HM metascore allows to stratify patients with excellent/intermediate/adverse PFS and OS with survival rates of 98% vs. 68% vs. 25%, respectively, after 5 years. In comparison to the rates by rISS of 86% vs. 65% vs. 40%, respectively, both groups with better as well as more adverse survival are delineated by the HM metascore.

## Additional files

Additional file 1: **Table S1.** PAM-based prediction error for light and heavy chain type as well as the sex of the patient. (PDF 1187 kb)

Additional file 2: **Figure S1.** Gene expression-based risk assessment as implemented in the GEP-R. **Figure S2.** Grouping myeloma into different subentities as implemented in the GEP-R. (PDF 1044 kb)

## Data Availability

The dataset supporting the conclusions of this article is available in the ArrayExpress repository under accession number E-MTAB-2299.
