# Supplementary figures and images for: Prospective target assessment and multimodal prediction of survival for personalized and risk-adapted treatment strategies in multiple myeloma in the GMMG-MM5 multicenter trial
Source: J Hematol Oncol. 2019 Jun 26;12:65. doi: 10.1186/s13045-019-0750-5 (PMC6595705; doi:10.1186/s13045-019-0750-5)

# OS GPI

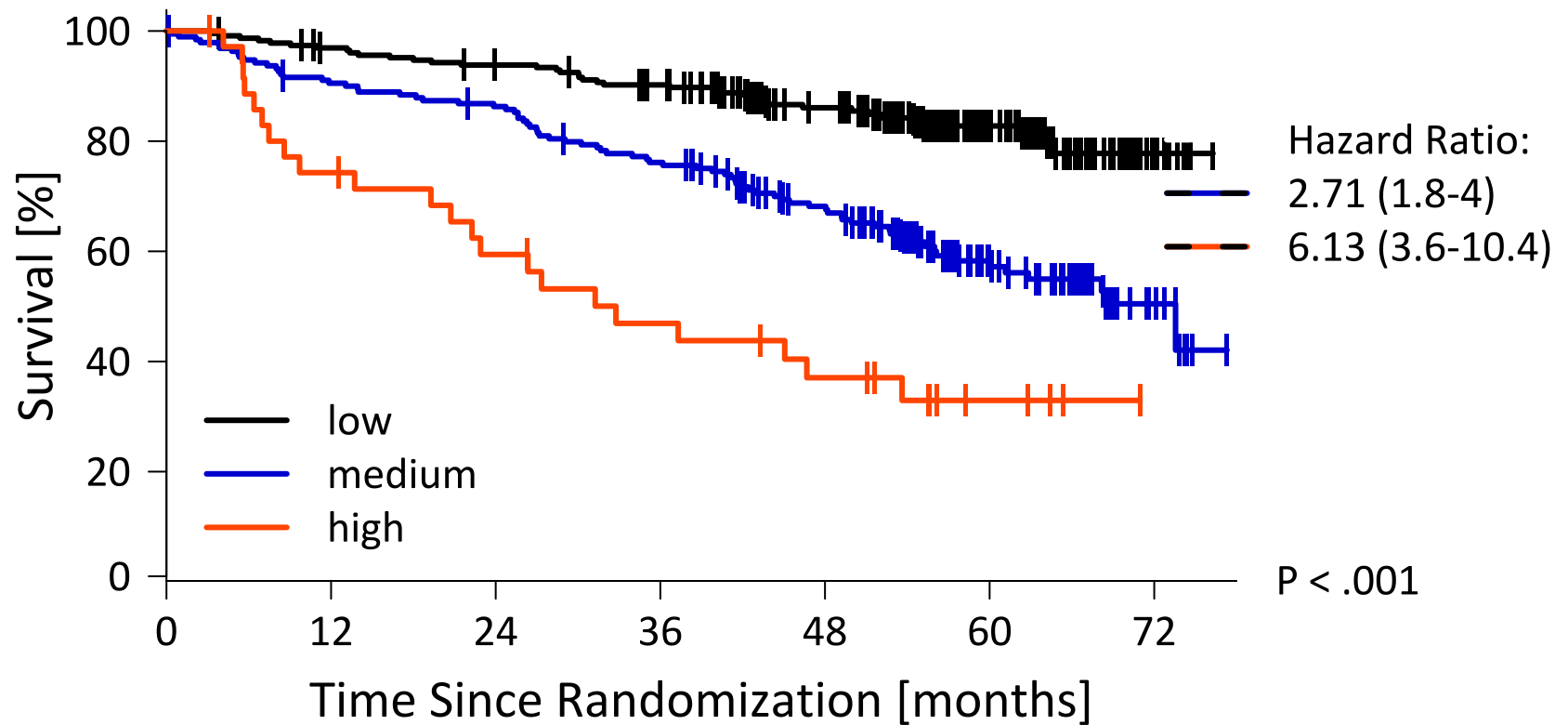

|     |     |     |     |     |    |    |        |
|-----|-----|-----|-----|-----|----|----|--------|
| 229 | 218 | 209 | 197 | 150 | 72 | 10 | low    |
| 191 | 171 | 162 | 142 | 113 | 55 | 9  | medium |
| 36  | 26  | 20  | 15  | 11  | 4  |    | high   |

Supplement: Supplementary file 1 — Table S1. PAM-based prediction error for light and heavy chain type as well as the sex of the patient. (PDF 1187 kb) [file 13045_2019_750_MOESM1_ESM.pdf]
