# Supplementary material for: Prospective target assessment and multimodal prediction of survival for personalized and risk-adapted treatment strategies in multiple myeloma in the GMMG-MM5 multicenter trial
Source: J Hematol Oncol. 2019 Jun 26;12:65. doi: 10.1186/s13045-019-0750-5 (PMC6595705; doi:10.1186/s13045-019-0750-5)
Supplement: Supplementary file 2 — Figure S1. Gene expression-based risk assessment as implemented in the GEP-R. Figure S2. Grouping myeloma into different subentities as implemented in the GEP-R. (PDF 1044 kb) [file 13045_2019_750_MOESM2_ESM.pdf]

Additional file 2

Figure S1. Gene expression-based risk assessment as implemented in the GEP-R.

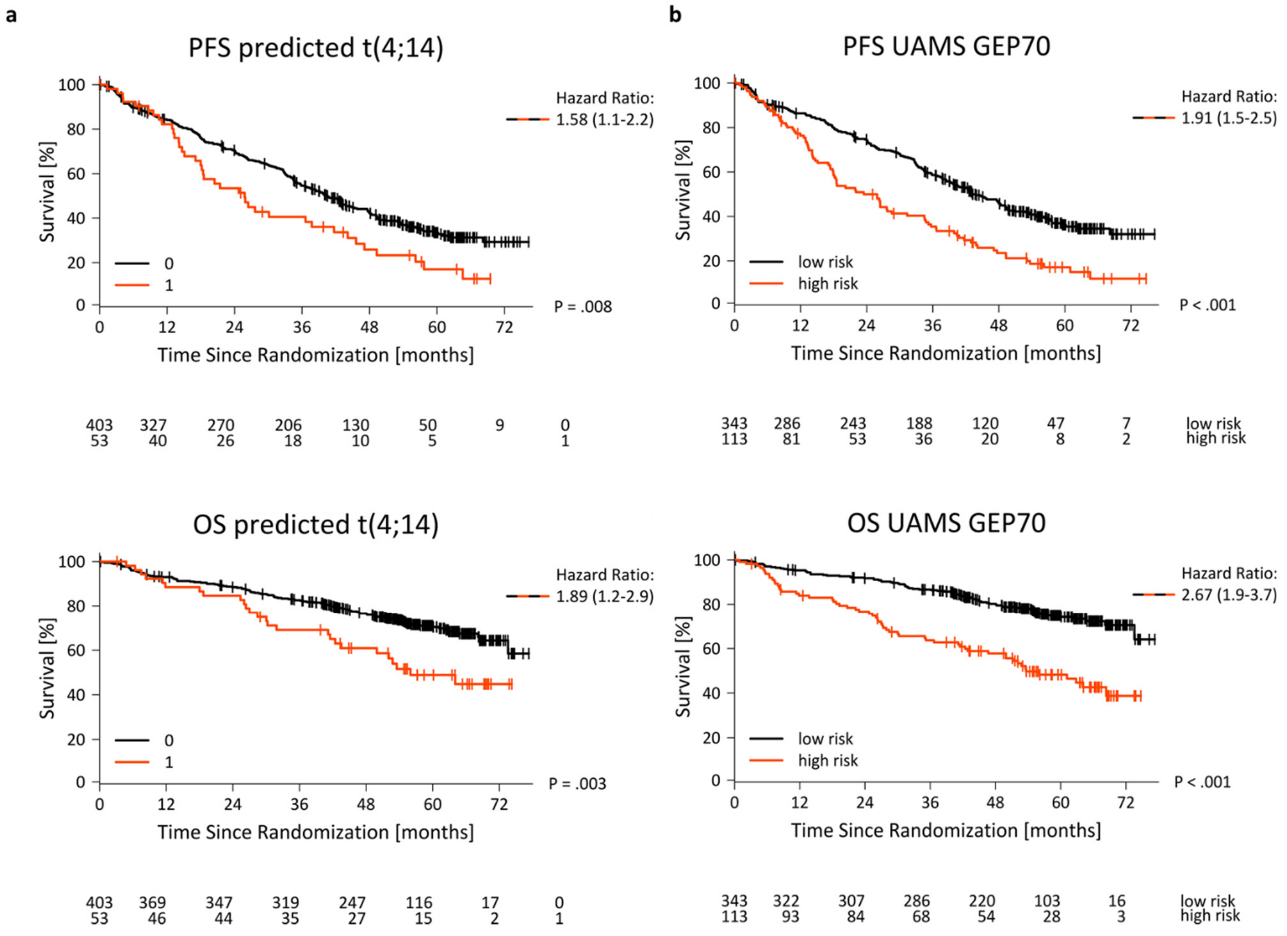

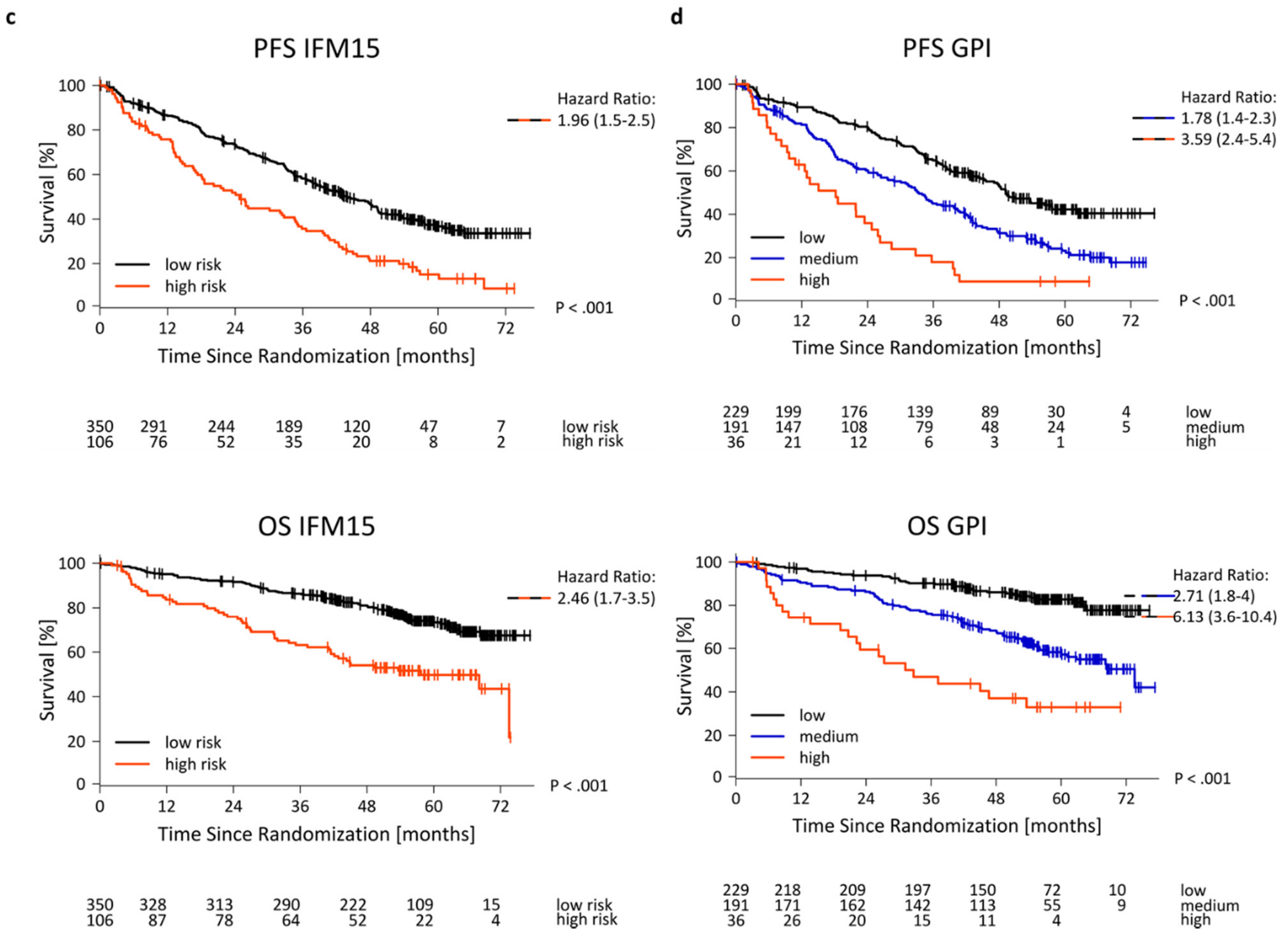

Shown are progression-free (PFS) and overall survival (OS) for (a) absence (0) vs. presence (1) of GEP-predicted t(4;14), (b) low vs. high risk according to GEP70-score, (c) low vs. high risk according to IFM15-score, as well as (D) GPI^{low} vs. GPI^{medium} vs. GPI^{high}.

Figure S2. Grouping myeloma into different subentities as implemented in the GEP-R.

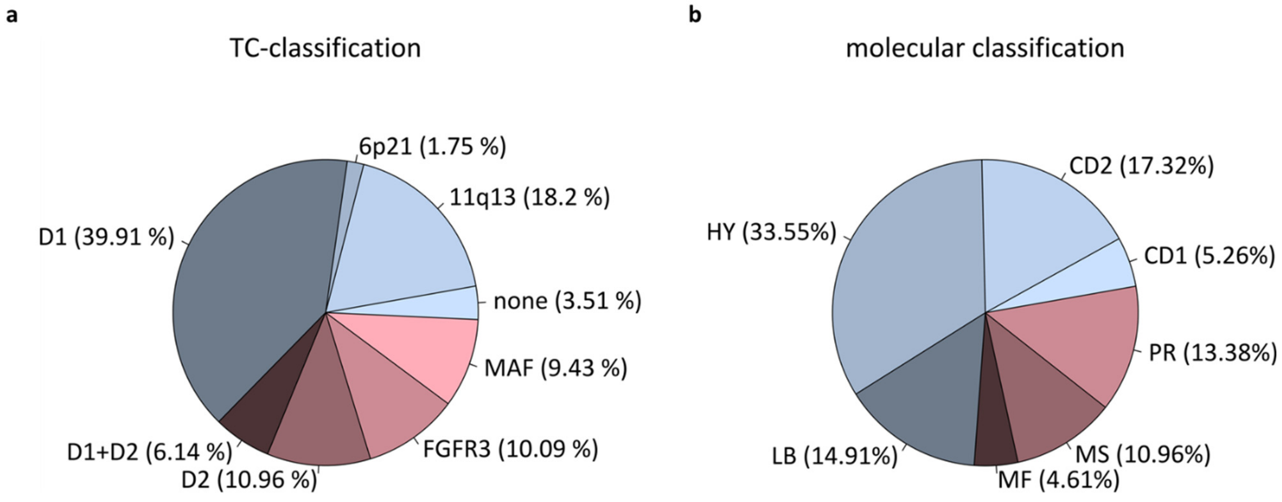

Shown are (a) the TC- and (b) the molecular classification of multiple myeloma. Percentages of patients in the different subgroups are given in brackets.
